# Simulation of CRISPR/Cas9-mediated gene editing for the *Vitellogenin* gene in *Apis mellifera*

**DOI:** 10.1038/s41598-026-57836-0

**Published:** 2026-06-17

**Authors:** Peymaneh Davoodi, Marzieh Atapour, Arezoo Shahsavari, Roozbeh Kiani

**Affiliations:** 1 Department of Animal Science, Faculty of Agriculture, University of Kurdistan, Sanandaj, Iran; 2 Department of Animal Science, Faculty of Agriculture, University of Kurdistan, Sanandaj, Iran; 3 Department of Animal Science, Faculty of Agriculture, University of Kurdistan, Sanandaj, Iran

**Keywords:** In silico CRISPR-CAS9, *Vitellogenin*, *Apis mellifera*, Omnigenic model, Gene editing, Biotechnology, Computational biology and bioinformatics, Genetics

## Abstract

CRISPR/Cas9 genome editing provides a powerful framework for interrogating gene function in *Apis mellifera*. Yet, empirical application remains challenging due to biological constraints, including haplodiploid genetics, narrow embryonic injection window, and the social rearing requirements that complicate functional validation. These constraints necessitate in silico pre-screening to maximize editing success before resource-intensive wet-lab implementation. Within the omnigenic framework, which distinguishes core regulatory genes from peripheral loci buffered by network effects, *vitellogenin* (*Vg*) represents an optimal target which is ancestrally dedicated to yolk provisioning; it has been co-opted to orchestrate diverse non-reproductive functions including longevity, stress resistance, immunity, and social behavior. We developed a computational pipeline to design a list of 57 and 56 candidate guide RNAs (gRNA) for targeted *Vg* knockout, evaluating candidate sites in both functional exons 2 and 3 based on structural accessibility and frameshift efficiency. Comparative analysis revealed complementary strengths in two top-best candidates from initial target pool of predicted gRNAs. The gRNA targeting exon 2 exhibits weaker secondary structure (ΔG = –0.25 kcal/mol versus –2.10 kcal/mol for exon 3), aligning with empirical evidence that sites with ΔG > –1.0 kcal/mol achieve 2–5 × higher Cas9 binding efficiency. This site yielded moderate frameshift frequency (77.8%; 61.9 percentile). Conversely, the predicted editing outcome for the gRNA targeting exon 3, despite stronger structural constraints, demonstrated superior functional disruption metrics demonstrating very high frameshift frequency (88.3%; 95.2 percentile), high *in silico* editing precision, minimal microhomology-mediated repair bias, and reproducible outcomes wherein nearly all predicted indels disrupt the coding sequence. Protein structure and domain analyses further predict that frameshift edits will generate a truncated protein missing all downstream functional domains. We recommend parallel empirical validation of both exon 2 and exon 3 targets to resolve the trade-off between structural accessibility (favoring higher editing rates) and frameshift efficacy (favoring complete loss-of-function). This dual-target strategy accommodates uncertainty in in vivo performance while maximizing the probability of generating informative phenotypes. Our *in silico* framework enables rational CRISPR design in non-model organisms by computationally balancing biophysical accessibility with functional impact, accelerating functional genomics in species where empirical optimization faces substantial biological constraints.

**Supplementary Information:**

The online version contains supplementary material available at https://doi.org/10.1038/s41598-026-57836-0.

## Introduction

The honeybees (*Apis mellifera*) are truly masterpiece of nature, essential pollinator species that plays a critical role in maintaining biodiversity and agricultural productivity worldwide. Beyond its ecological and economic importance, honeybees display complex colony-level traits such as caste differentiation and division of labor, which are central to the organization and resilience of social societies as a model organism for studying social behavior^1,2^. However, the mechanistic basis of these traits remains incompletely understood, underscoring the need for molecular-level investigations that can link gene function to social behavior. Central to these traits is the *vitellogenin* (*Vg*) gene, a highly conserved protein, encoding a multifunctional yolk precursor protein traditionally associated with reproduction but now recognized for its broader physiological roles^3–9^.

*Vg* influences immunity, oxidative stress resistance, and division of labor within colonies, thereby acting as a molecular link between reproduction and social organization^4,7,10–12^. The *Vg’s* multifunctionality makes it a powerful target for studying how genes influence complex traits in social insects. Despite extensive association studies, the alteration of *Vg* via gene editing has not been systematically investigated and remained challenging.

The advent of CRISPR/Cas9 genome editing technology has revolutionized functional genomics by enabling precise and programmable platform for targeted modifications of specific gene or genes in human and productive animals^13,14^. Recent advances have demonstrated the feasibility of CRISPR/Cas9-mediated gene editing in *Apis mellifera*. For example, Sinakevitch et al.^15^ applied CRISPR/Cas9 to examine antibody specificity against the insect GABAA receptor subunit RDL and the metabotropic glutamate receptor mGluRA in honeybee brain sections^15^. Hu et al.^16^ and Kohno et al.^17^ achieved high-efficiency CRISPR/Cas9-mediated editing in honeybee embryos, targeting the *Mrjp1* and *Pax6* genes, with biallelic knockout rates exceeding 70%^16,17^. Wagner et al.^18^ reported highly efficient site-specific integration of DNA fragments into the honeybee genome by editing the *dsx* gene^18^. More recently, a novel liposome-mediated CRISPR/Cas9 delivery system via drone sperm targeting the *JHAMT* gene, reported achieving efficient heritable knockouts and offering a scalable alternative to embryo microinjection for advancing honeybee genetics and health^19^.

Collectively, these studies highlight the potential of CRISPR/Cas9 for functional genomics in social insects. However, in vivo editing in *Apis mellifera* is limited by developmental complexity, off-target risks, and ethical/logistical barriers, difficulty of embryo microinjection, limited survival rates of edited individuals^16,20,21^ motivating rigorous in silico evaluation. In silico approaches offer a practical solution, allowing researchers to design and simulate studies without costly lab resources^22–27^.

Although vast studies exist for introducing and comparing in silico CRISPR tools^27–29^, and despite *vitellogenin*’s well-documented pleiotropic roles in honey bee physiology and social behavior, the molecular mechanisms underpinning its diverse functionalities remain incompletely characterized. Structural insights into its multidomain architecture, particularly the lipid-binding cavity responsible for yolk precursor transport and the functionally enigmatic von Willebrand factor type D domain, have only recently begun to emerge through cryo‑EM analyses of insect’s *Vg*^8^. Also, the precise contributions of these domains to *Vg*’s non‑reproductive functions (e.g., antioxidant activity, immune modulation, and behavioral regulation) await detailed mechanistic elucidation.

The aim of this study was implementing comprehensive pure in silico framework for CRISPR/Cas9-mediated editing of the *vitellogenin* gene in *Apis mellifera*. By combining sequence analysis, target site prediction, and off-target evaluation, we aim to establish a robust computational pipeline that facilitates future experimental validation. This work not only advances our understanding of *vitellogenin*’s role in *Apis mellifera* biology but also underscores the potential of computational gene-editing approaches in addressing challenges in pollinator health and conservation.

## Materials and methods

### Target gene selection

The integral step in any CRISPR/Cas9 experiment is the careful selection of the target gene. Candidate target gene of *Vg* were prioritized based on standard principle through vast systematic review of biological relevance^30^ functional domain targeting^31^, with pleiotropic functions^6^, conservation and expression criteria^32,33^. *Vg* expression is highly plastic which, exhibits remarkably complex tissue-specific expression patterns in honey bees that extend well beyond its canonical reproductive role. Therefore, temporal sampling is critical when comparing tissues across behavioral states or developmental stages. The abdominal fat body serves as the principal site of *Vg* transcription and *Vg* protein synthesis, functioning analogously to the vertebrate liver^34^. Expression in this tissue demonstrates pronounced caste and behavioral specificity *Vg* known as a queen caste molecular signature, while among workers, nurse bees maintain substantially higher expression than foragers, with levels declining during the nurse-to-forager transition^34,35^. Recent advances in single-cell transcriptomics have revealed unexpected complexity in neural *Vg* expression. Zhang et al.^36^ demonstrated that *Vg* is highly expressed specifically in ensheathing glial cells of queen brains, but not in neurons or other glial subtypes, proposing this pattern as a molecular signature of caste differentiation. This brain-localized *Vg* may influence behavioral maturation, aging, and social organization through neuroendocrine signaling pathways^36^.

In reproductive tissues, *Vg* expression patterns diverge markedly between castes. Queen ovaries show robust expression consistent with canonical yolk precursor function during oogenesis^35,37–40^. In contrast, worker ovaries, historically thought to lack *Vg* expression, exhibit low but detectable transcript levels that increase significantly following royal jelly feeding and correlate with ovary activation state, even in functionally sterile individuals^41^, (Cardoso‐Júnior et al. 2021). The midgut presents a particularly intriguing case of tissue-specific *Vg* localization. Immunohistochemical analyses combined with RT-qPCR revealed abundant *Vg* protein throughout enterocytes of both nurses and foragers, yet *Vg* transcript levels remain low, suggesting that *Vg* is imported from hemolymph rather than locally synthesized^42^. Notably, nurses exhibit greater *Vg* concentration near the basement membrane, regenerative crypts, and peritrophic membrane compared to foragers, implying behavior-specific functional roles possibly related to antimicrobial defense or antioxidant protection^42^. Circulating *Vg* in hemolymph represents a substantial protein pool, constituting up to 70% of total hemolymph protein in queens^9^. This circulating fraction functions as a nutrient reservoir, antioxidant, and immune modulator, with titers correlating strongly with behavioral state, high in nurses, low in foragers, and experimental knockdown triggering precocious foraging onset^9,43^. Additional tissues with documented *Vg* protein localization include hypopharyngeal glands, where *Vg* is incorporated into royal jelly synthesis^4,39,44^, head fat bodies surrounding the brain, anatomically distinct from abdominal fat deposits^45^; and muscle tissue^46^.

### Gene sequence retrieval

The complete nucleotide sequence of the *vitellogenin* (*Vg*) gene in *Apis mellifera* was retrieved from the NCBI database (Gene ID: 406,088- Accession ID: NC_037641.1). The FASTA format sequence was downloaded and used as the reference template for all computational analyses^47^.

### Potential guide RNAs and target sites

Potential CRISPR/Cas9 target sites within the *Vg* gene were predicted using guide RNA (gRNA) design platform of CHOPCHOP v3 (https://chopchop.cbu.uib.no/) selected for its native integration of the Amel_HAv3.1 reference genome, transparent on-target efficiency predictions, and comprehensive off-target scoring algorithms. For *Apis mellifera*, CHOPCHOP is especially useful due to flexible genome support.

Also, alternative design platforms (CRISPOR, E-CRISP Design, and Cas-OFFinder) were initially evaluated but excluded due to incomplete *Apis mellifera* genome annotation, lack of native *A. mellifera* support, or unreliable target sequence retrieval. To ensure high specificity, all candidate gRNAs were validated via BLASTn against the NCBI *A. mellifera* reference genome assembly (GCF_003254395.2), and any sequences exhibiting significant off-target homology were excluded prior to synthesis.

CHOPCHOP parameters were set for SpCas9 nuclease with the canonical PAM sequence NGG. Then candidate guide RNAs (gRNAs) were selected based on: GC content between 40–60%, target site length of 20 nucleotides and minimum of self-complementarity to avoid secondary structure formation^48,49^.

### Off-target prediction

To minimize unintended genomic modifications, off-target potential for each candidate guide RNA was evaluated using the integrated prediction algorithm in CHOPCHOP v3^48^. Candidate 20-nt protospacer sequences and their adjacent protospacer-adjacent motifs (PAMs) were exhaustively aligned against the *Apis mellifera* reference genome assembly (Amel_HAv3.1) using the Bowtie2 alignment engine. The pipeline was configured to permit up to three mismatches and zero insertions or deletions, consistent with empirical evidence indicating that SpCas9 activity declines precipitously beyond three mismatches, particularly within the 3′-PAM-proximal seed regions^31^.

### Ranking of gRNAs

Design of guide RNA (gRNA) with high efficiency and specificity is vital for successful application of the CRISPR gene editing technology^21^.

### Primer design

To validate CRISPR-Cas9-mediated modifications of the *vitellogenin* (*Vg*) gene, five primer pairs were designed to flank the primary gRNA target site, specifically amplifying genomic regions encompassing the predicted Cas9 cleavage loci within selected exons 2 and 3^48,50^.

Comprehensive in silico validation was performed against the *Apis mellifera* reference genome assembly (Amel_HAv3.1) using NCBI Primer-BLAST (https://www.ncbi.nlm.nih.gov/tools/primer-blast/) and the UCSC In-Silico PCR tool (https://genome.ucsc.edu/cgi-bin/hgPcr) to confirm locus specificity and exclude cross-amplification with paralogous sequences or repetitive elements. Thermodynamic properties were further evaluated using IDT OligoAnalyzer^51^, with primers optimized for uniform melting temperatures, minimal self- and hetero-dimerization potential, and negligible secondary structure formation. All primer sets were screened to ensure compatibility with standard two-step PCR cycling conditions. In silico analyses confirmed that each primer pair (detailed in Table 2) exhibits high specificity for the *Vg* locus, with no predicted off-target binding sites or primer-dimer artifacts under standard amplification conditions.

### Secondary structure of predicted gRNAs

The secondary structure of gRNAs was predicted using RNAfold (ViennaRNA package) to evaluate potential hairpin formation and structural stability. Thermodynamic binding energy between gRNA and target DNA was calculated to assess editing efficiency to ensure efficient Cas9-gRNA complex formation^52–55^.

### Simulating CRISPR/Cas9-mediated editing outcomes

To computational evaluation of the mutational consequences of CRISPR/Cas9-mediated editing of the *vitellogenin* (*Vg*) gene in *Apis mellifera*, predicted insertion and deletion (indel) profiles were generated using inDelphi, which employs a machine learning framework to predict how DNA repairs itself following Cas9-induced double-strand breaks, specifically modeling insertions and deletions generated through non-homologous end joining (NHEJ), the predominant repair pathway in CRISPR editing. This approach provides insight into the likely functional consequences of targeted gene disruption^29^.

### Functional domain mapping

SMART (Simple Modular Architecture Research Tool) a web resource was used for the of *Vg* domains identification and architectures before and after in silico editing^56–59^.

### Protein structure prediction

To in silico evaluation of the structural consequences of CRISPR-Cas9-mediated modifications, three-dimensional (3D) protein models of both wild-type and in silico-edited Vg were generated using complementary computational approaches. Homology-based modeling was performed with SWISS-MODEL, which executed a standardized pipeline comprising template identification via sequence homology searches, alignment-guided backbone modeling, loop region reconstruction, and side-chain optimization. To independently corroborate these predictions and assess folding robustness in the absence of high-similarity templates, de novo structure prediction was conducted using AlphaFold2 for both the wild-type and edited sequences. Comparative structural analyses were then performed to evaluate conformational shifts, domain stability, and potential functional perturbations introduced by the targeted edits. Model quality and stereochemical validity were assessed using predicted Local Distance Difference Test (pLDDT) scores to verify backbone geometry^11,25,60^ (Davoodi 2023).

## Results

### Target exons selection rationale

Therefore, exons 2 and 3 were prioritized for CRISPR/Cas9 targeting based on functional domain mapping, positional efficiency for loss-of-function induction, and the feasibility of gRNA design. Exon 1 was excluded due to its extremely short length (27 bp; 5′-ATGTTGCTACTTCTAACGCTTTTACTGTTCG-3′), high GC content (~ 63%), and immediate proximity to the start codon factors that collectively obstruct efficient gRNA design and risk alternative translation initiation, yielding hypomorphic isoforms. In contrast, exons 2 and 3 reside within the first ~ 15% of the coding sequence, ensuring that frameshift indels efficiently trigger nonsense-mediated decay and produce severely truncated polypeptides. Functionally, exon 2 encodes the lipid-binding cavity of the N-terminal β-sheet, while exon 3 contains the N-terminal portion of the von Willebrand factor type D (VWD) domain; disruption of either module disrupts distinct aspects of *Vg*’s dual role in lipid transport and immune recognition. This strategy maximizes the probability of generating a true null allele while enabling modular phenotypic dissection.

**Fig. 1:**
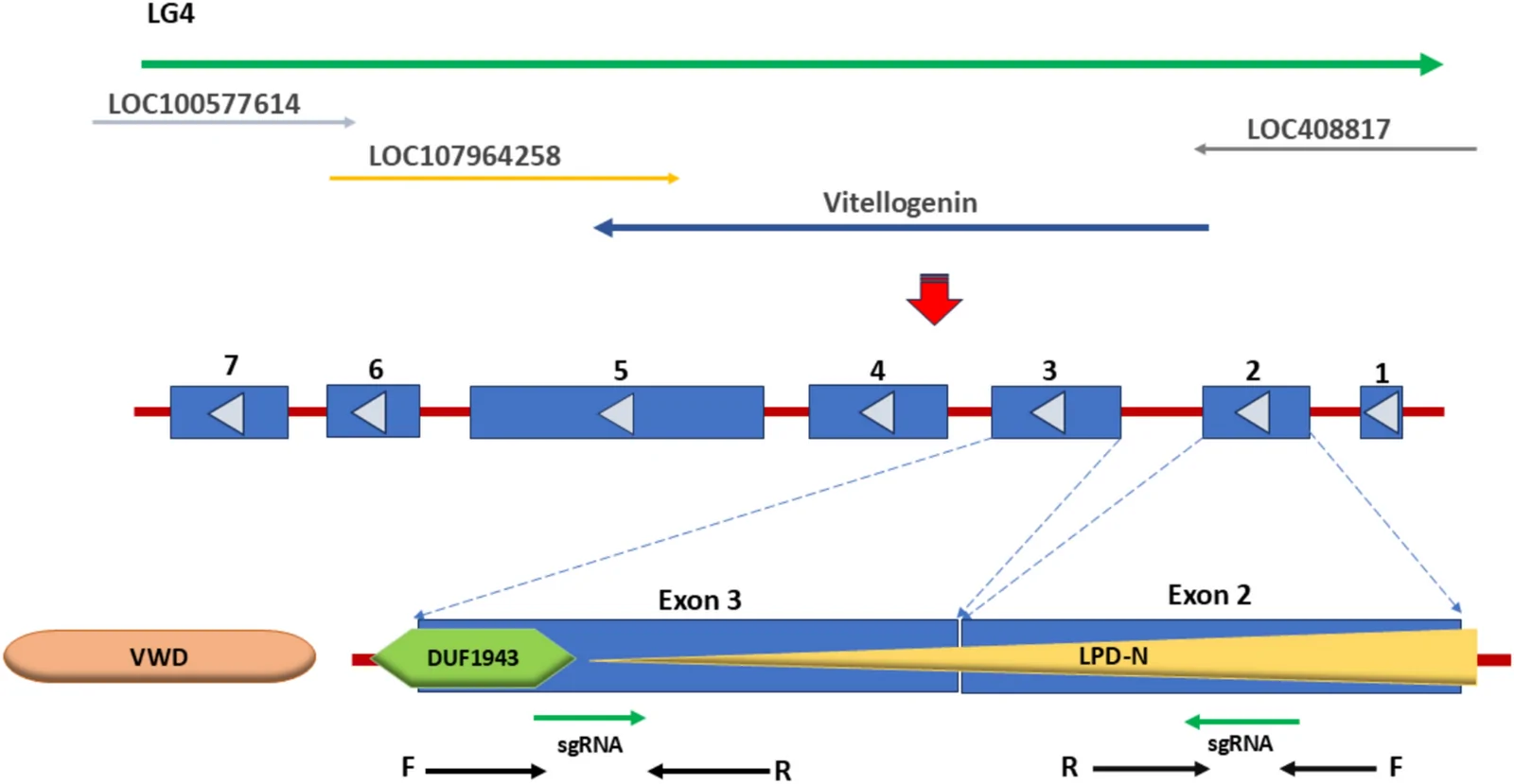
Schematic representation of the target gene architecture and CRISPR/Cas9 editing strategy focused on exons 2 and 3.

Schematic representation of the target gene architecture and CRISPR/Cas9 editing strategy focused on exons 2 and 3. The upper panel shows the genomic context of the vitellogenin locus and neighboring genes (LG4, LOC100577614, LOC107964258), with arrows indicating transcriptional orientation. The central panel illustrates the seven-exon organization, with the red arrow denoting the intended editing window. The expanded lower panel details the conserved functional domains within exons 2 and 3, as identified by SMART analysis: the VWD domain, DUF1943, and the LPD-N domain. Green arrows mark the binding sites of the designed single-guide RNAs (sgRNAs) for targeted mutagenesis, while black arrows (F/R) indicate the positions of forward and reverse primers used for PCR-based validation. This structural overview substantiates the rationale for selecting exons 2 and 3 as optimal targets for inducing functional gene disruption (Fig. 1).

### Top Guide RNAs and In silico validation of targeting exons 2 and 3

**Fig. 2:**
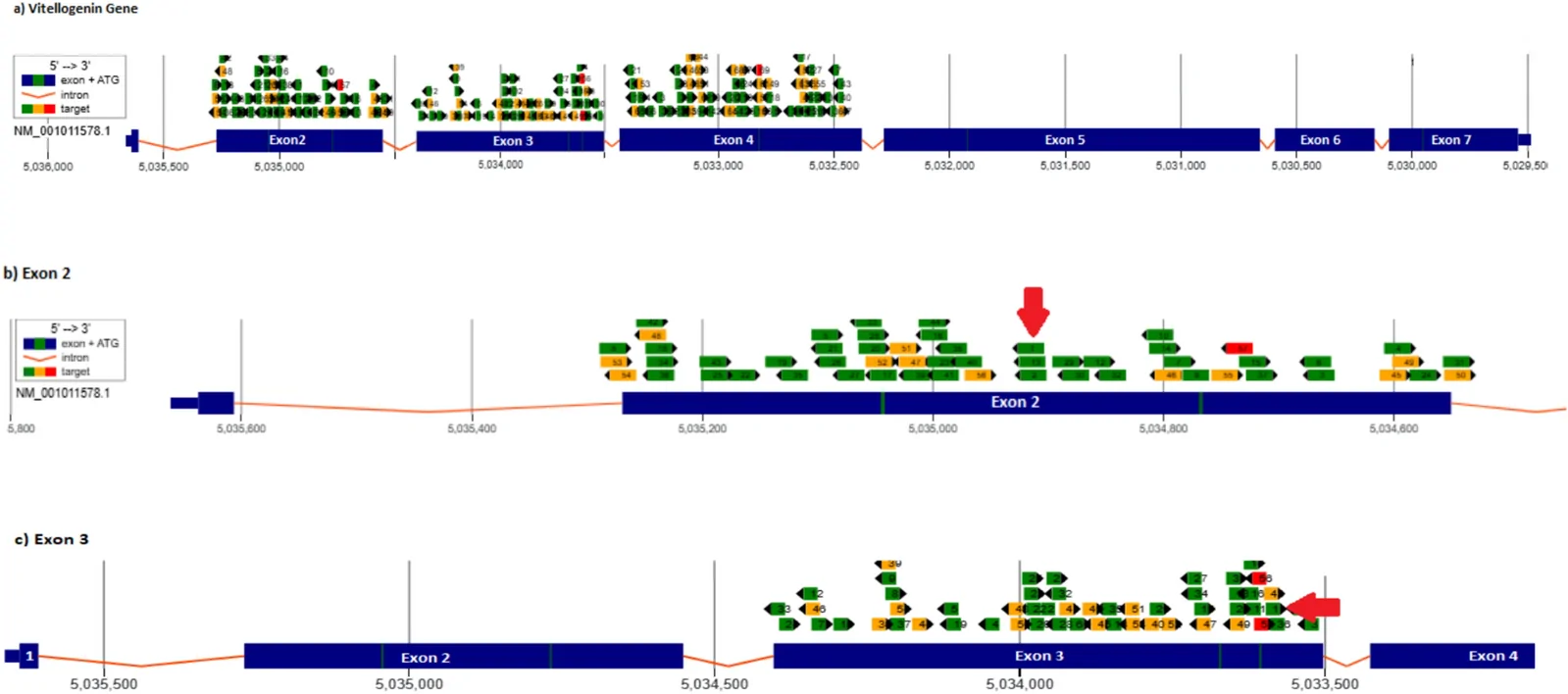
The graph of predicted Guide RNAs targeting exon 2 and exon 3 of *vitellogenin* gene in honeybee.

Guide RNAs (gRNAs) targeting the *vitellogenin* (*Vg*) gene were designed using the CHOPCHOP web tool and evaluated under an embryonic stem cell context (mouse ESCs recommended for pluripotency and genetic tractability) (Fig. 2a). The presence and positions of protospacer adjacent motif (PAM) sites within the first two examined exons were manually confirmed to ensure Cas9 compatibility.

The top-ranked gRNA for exon 2 GTATGTTCACCTCCCAAGTG for (target site sequence of GTATGTTCACCTCCCAAGTGGGG; genomic coordinate NC_037641.1:5,034,904, + strand) exhibited GC content 50%, no predicted hairpin or dimer formation, off-target matches limited to MM0–MM3, and a predicted editing efficiency of 69.43%. These results were cross-validated by BLAST against the *Apis mellifera* genome to exclude significant unintended homology, yielding a high-confidence candidate for downstream analysis (Fig. 2b, Table 1).

**Table 1:** Top ranked predicted gRNAs targeting exon 2 and exon 3 of *vitellogenin* gene in honeybee.

Exon	Target sequence	Genomic location	Strand	GC content (%)	Self-comp	MM 0–3	Efficiency
2	GTATGTTCACCTCCCAAGTG**GGG**	NC_037641.1:5,034,904	+	50	0	0	69.43%
3	GGGGACTACCAAAAGCATGG**AGG**	NC_037641.1:5,033,574	-	55	0	0	74.83%

Furthermore, a parallel design and evaluation pipeline was applied to exon 3. Candidate gRNAs were prioritized by specificity scores, predicted editing efficiency, and mutational outcome profiles. The selected gRNA targeting exon 3 of GGGGACTACCAAAAGCATGG for (target site sequence: GGGGACTACCAAAAGCATGGAGG; genomic coordinates NC_037641.1:5,033,574, reverse strand) demonstrated GC content 55%, no predicted hairpin or dimer formation, off-target matches restricted to MM0–MM3, and a predicted efficiency of 74.83%. BLAST screening confirmed absence of substantial homology to non-target genomic regions. This in silico filtering produced high-specificity gRNA candidates suitable for CRISPR/Cas9-mediated disruption of key exonic regions of the *Vg* gene (Fig. 2c, Table 1).

### Primer design

Five primer pairs targeting the *Vg* locus were successfully designed to flank the primary CRISPR-Cas9 gRNA cleavage site within exons 2 and 3. In silico specificity screening against the *Apis mellifera* reference genome (Amel_HAv3.1) using NCBI Primer-BLAST and UCSC In-Silico PCR confirmed that all primer pairs uniquely amplify the intended *Vg* region, with no predicted off-target binding to paralogous sequences, repetitive elements, or other genomic loci. Thermodynamic analysis via IDT OligoAnalyzer indicated that all primers exhibited uniform melting temperatures (Tm range 59–61°C), minimal self-complementarity (ΔG >  − 5.38 kcal/mol), and negligible potential for hairpin or hetero-dimer formation under standard PCR conditions. Predicted amplicon lengths ranged from 245 to 271 bp, optimizing resolution for agarose gel electrophoresis and Sanger sequencing. Collectively, these in silico validations confirmed the suitability of some of primer sets (Table 2) for specific, efficient amplification of the *Vg* target region, enabling reliable downstream detection of Cas9-induced indels.

**Table 2:** Five primer pairs for target sites in exons 2 and 3 of *vitellogenin.*

	Pair	Left primer	LP Tm	Right primer	RP Tm	Amplicons	Hairpin	Self dimer
Exon2	1	CAATATGCTCGTGTCCAACAGT	60.1	TTCACTTTGAGTGTCCACTTGC	60.3	222	-0.36	-3.91
	2	GGTATTCTGATAAAGGCGTTGC	60.0	TTCACTTTGAGTGTCCACTTGC	60.3	297	0.03	-3.61
	3	AATATGCTCGTGTCCAACAGTC	59.1	TTCACTTTGAGTGTCCACTTGC	60.3	221	-0.36	-3.91
	4	CGGTATTCTGATAAAGGCGTTG	60.8	TTCACTTTGAGTGTCCACTTGC	60.3	298	0.03	-3.61
	5	CAGATCAGGATGAAACACGGT	60.0	ATCGTAGAGAACCTCGCATTTC	59.8	228	-0.32	-3.91
Exon 3	1	TACATGGACGGTGTTCAGAGAC	60.0	TCTGATGTAGCTGTCAGTCGGT	59.9	166	-1.11	-5.38
	2	CACCATGAACAGGAAGCAAATA	60.0	TCTGATGTAGCTGTCAGTCGGT	59.9	265	-0.83	-5.38
	3	ACAAGTCTCAGGTTGTCAAGCA	60.0	TCTGATGTAGCTGTCAGTCGGT	59.9	191	-0.48	-5.24
	4	AGCAAATAAGCGAGTTGGAGAG	60.0	TCTGATGTAGCTGTCAGTCGGT	59.9	251	-0.41	-3.9
	5	TACATGGACGGTGTTCAGAGAC	60.0	GCTCGTTGCTCACTTTAGGATT	59.9	286	-0.89	-3.61

### Secondary structure and thermodynamic stability of gRNAs

To facilitate precise in silico CRISPR-Cas9 targeting of the *vitellogenin* gene, the ViennaRNA web service, a specialized bioinformatics platform designed for structure prediction was used.

The ViennaRNA streamlines the gRNA design process and reducing manual errors by prototyping of gRNAs before committing to costly wet-lab experiments which supports insect genome editing, which often lacks robust annotation compared to mammalian systems.

The thermodynamic ensemble prediction for the designed gRNAs revealed free energy kcal/mol, indicating a relatively stable folding conformation. The minimum free energy (MFE) structure of the ensemble population, suggesting a dominant structural arrangement that may favor consistent functionality. An ensemble diversity score reflecting amount of variation among possible structures, highlighting some degree of flexibility without compromising core folding features.

**Fig. 3:**
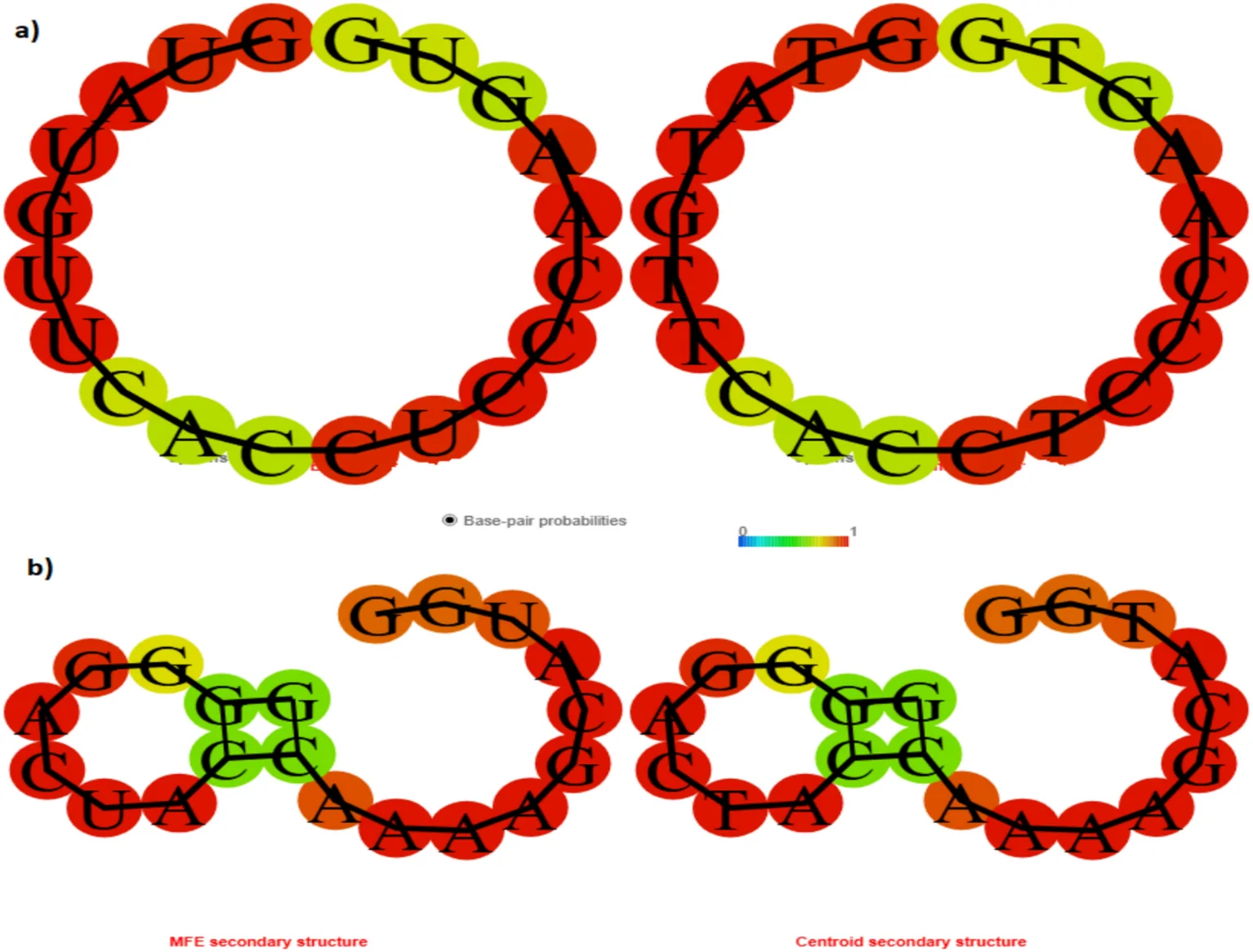
MFE and centroid secondary structures of first ranked gRNAs targeting exons 2 (**a**) and 3 (**b**). Stems (Helices) are indicating regions of stable internal pairing, potentially relevant to gRNA stability and recognition. Black pairing lines indicate predicted hydrogen bonds (base pairs). The centroid Secondary structure represents the most probable RNA folding pattern within the thermodynamic ensemble.

Loops and bulges are common in gRNAs due to their designed structure important for function and interaction with Cas proteins (Fig. 3).

The predicted free energy of the thermodynamic ensemble in targeted site of exon2 is -0.25 kcal/mol. The frequency of the MFE structure in the ensemble and its diversity are 66.28 and 1.45 respectively. While the predicted free energy of the thermodynamic ensemble in targeted site of exon 3 is -2.10 kcal/mol. The frequency of the MFE structure in the ensemble and its diversity are 61.88% and 2.26 respectively.

**Fig. 4:**
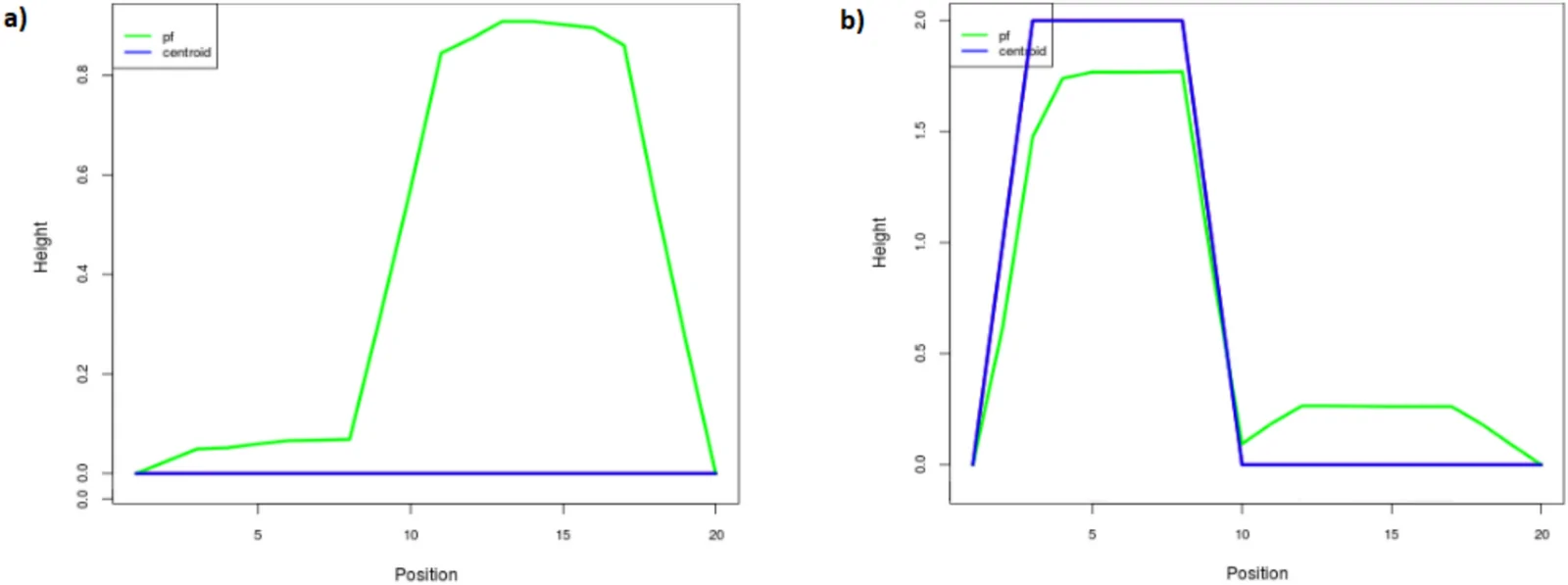
Montaine plots of two first ranked gRNAs targeting exons 2 and 3.

In Mountain Plots (Fig. 4), PF (Partition Function) curves reflect the ensemble of all possible structures, weighted by their thermodynamic probabilities. Providing insight into structural variability and confidence in base-pair predictions. A flatter PF curve suggests less structural certainty, while sharp features indicate dominant motifs.

Centroid Curve represents the structure with minimum average base-pair distance to all other structures in the ensemble. Balances between stability and representativeness was used to validate MFE predictions or highlight alternative folding possibilities.

### Simulating CRISPR/Cas9-mediated editing outcomes

It was predicted that after CRISPR/SpCas9 induced a double-strand break (DSB) in the Vg gene, the cell would repair the damage primarily through non-homologous end joining (NHEJ)^61^. These predictions help us understand how the gene might be disrupted after editing. High-frequency indels near functional domains increase the chance of loss-of-function mutations. According to inDelphi results, most of the predicted indels at our target site in exon 2 and 3 cause frameshifts, which disrupt the reading frame of the *Vg* gene. This increases the likelihood of producing a nonfunctional protein, which is exactly what we aim for in a knockout experiment specially in targeted site of exon 3.

**Fig. 5:**
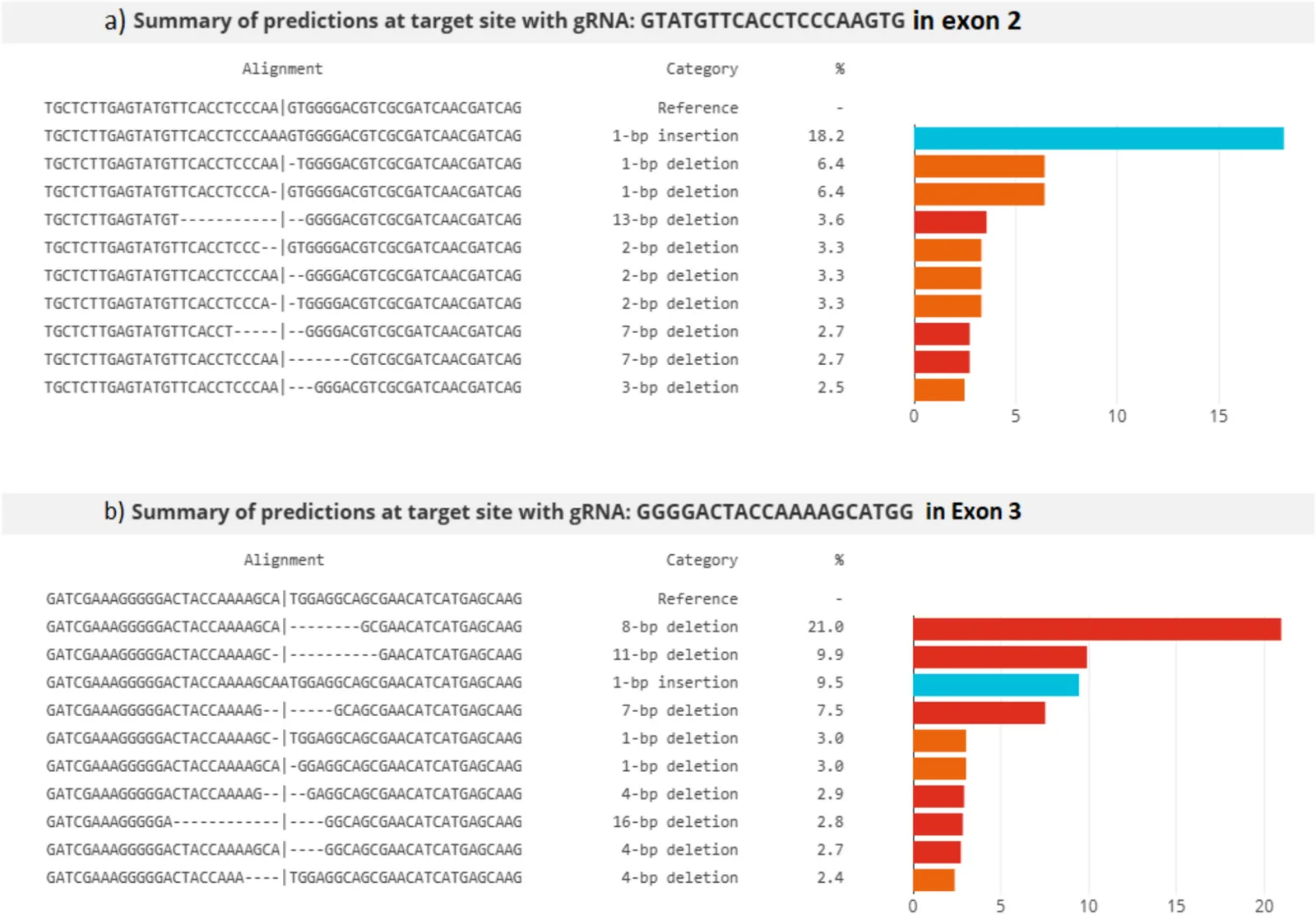
Summary of the predicted editing outcomes for at first-ranked the gRNA targeting exons 2 and 3.

inDelphi provides a distribution of indel sizes, showing how many bases are likely to be inserted or deleted in both target sites of exons 2 and 3. Most common indels in exons 2 and 3 were, 1bp insertion with 18.2% and 8bp deletion with 21% frequency respectively (Fig. 5). These small changes can significantly affect gene function depending on their position and frame shift.

**Fig. 6:**
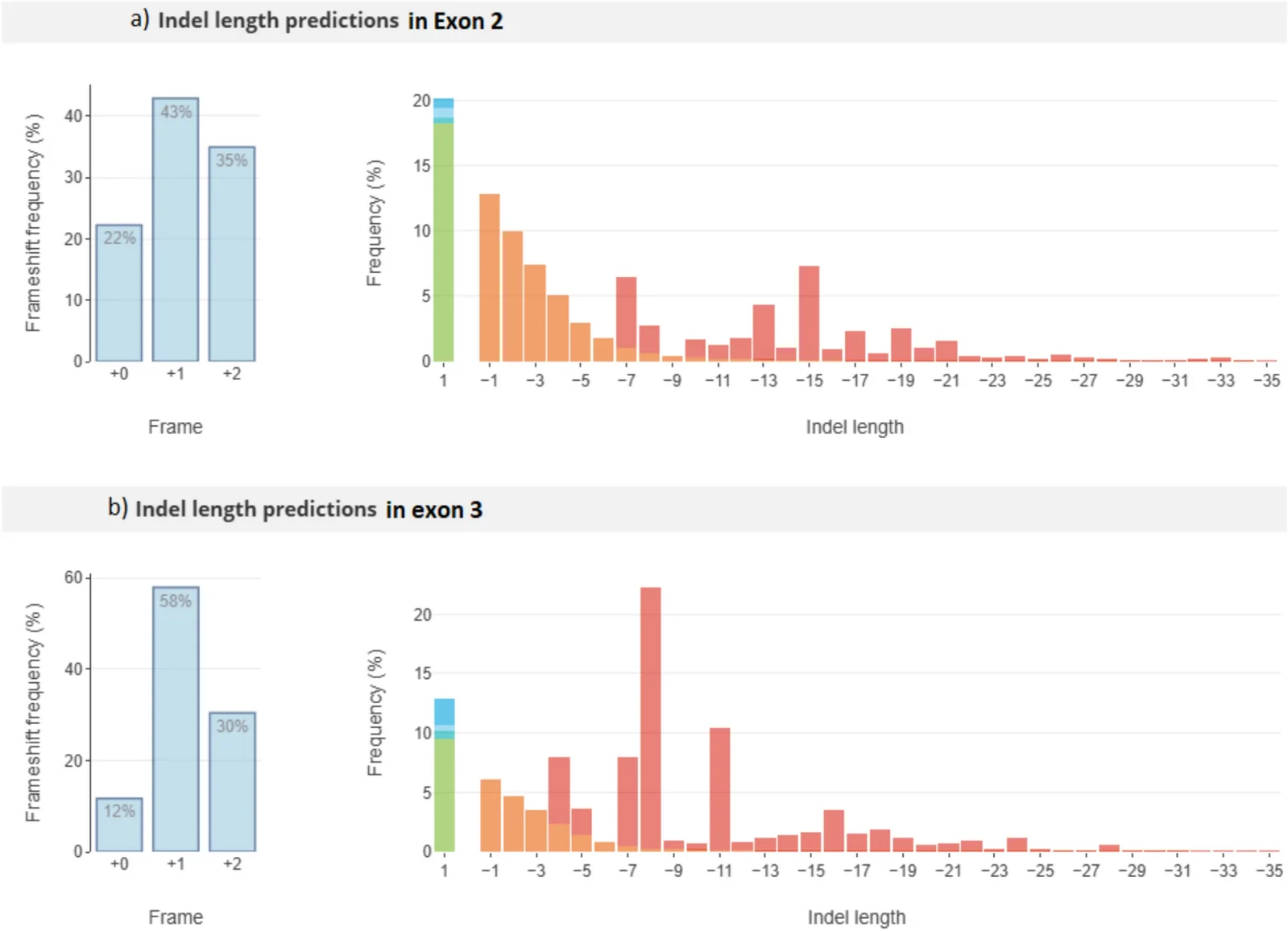
Indel length prediction and frame shift frequencies at first-ranked target sites in exons 2 (**a**) and 3 (**b**). 0 (in-frame) produces No shift so that codons remain aligned. + 1 (frameshift): One base added or removed; codons misalign. + 2 (frameshift): Two bases added or removed; codons misalign differently.

Indel profiling of the top-ranked target sites revealed that frameshift mutations predominated in both exons, with ± 1 bp indels representing the most frequent class of editing outcomes, followed by ± 2 bp indels. Non-frameshift (in-frame) alterations, comprising indels divisible by three nucleotides, accounted for 22% of predicted edits at the exon 2 target site and 12% at the exon 3 target site (Fig. 6). This distribution indicates that 78% and 88% of predicted editing events in exons 2 and 3, respectively, would disrupt the *vitellogenin* open reading frame, thereby favoring complete loss-of-function phenotypes.

**Fig. 7:**
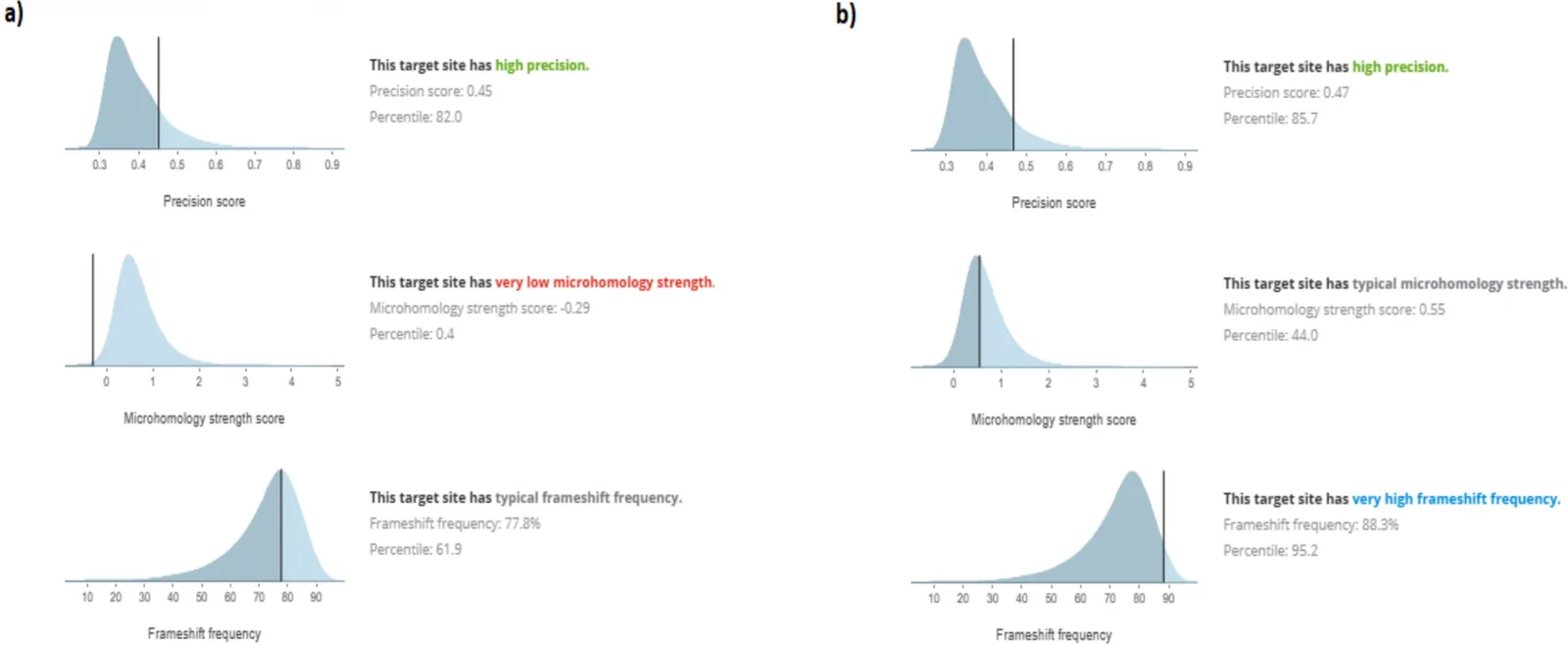
Comparison prediction edit precision, microhomology strength and frameshift frequency at first-ranked target sites in exons 2 (**a**) and 3 (**b**).

Precision score of 0.45 (82.0 percentile) in first-ranked target site of exon 2 and, 0.47 (85.7) in first-ranked target site of exon 3 are predicted. These high scores mean the repair outcomes at both sites are highly predictable. In other words, the same types of indels are likely to occur repeatedly, which is ideal for consistent gene editing results. And this precision score is a bit higher in target site in exon 3 (Fig. 7).

In addition, microhomology score of 0.29 (0.4 percentile) in first-ranked target site of exon 2 and, 0.55 (44.0) in first-ranked target site of exon 3 are predicted. In first-ranked target site of exon 2 is low which indicates weak microhomology at the cut site. Microhomology refers to short matching sequences near the break that can guide repair. A low score suggests that microhomology-mediated end joining (MMEJ) is unlikely, meaning the repair will mostly occur through classical NHEJ, which is more random (Fig. 7).

Meanwhile for first-ranked target site of exon 3 the microhomology score is typical which indicates moderate microhomology at the cut site of exon 3. This moderate score suggests that MMEJ can occur in more than half of repairs.

Furthermore, frameshift frequency of 77.8% (61.9 percentile) for first-ranked target site of exon 2 and, 88.3% (95.2) in first-ranked target site of exon 3 are estimated (Fig. 7).

### Functional domain map

Domain architecture analysis of *Apis mellifera vitellogenin* was performed using the SMART to identify evolutionarily conserved functional domains and represent their precise boundaries within the protein sequence. This analysis revealed three principal domains, including the N-terminal lipoprotein domain (LPD_N), a domain of unknown function (DUF1943), and a von Willebrand factor type D domain (VWD) (Table 3). The LPD_N domain, which mediates lipid binding and represents the functionally critical region of *vitellogenin*, spans amino acid residues 22–741. Notably, both exon 2 (amino-acid positions 11–250 of the coding sequence) and exon 3 (amino-acid positions 251–550) segments are entirely embedded within this LPD_N domain. Targeting certain regions of exons 2 and 3 of the *Vg* gene was strategically selected to maximize the probability of generating disruptive frameshift mutations within the structurally and functionally indispensable lipoprotein domain. Sequence analysis of in silico editing confirmed that Cas9-induced indels at this early genomic position resulted in a downstream shift of the translational reading frame. This frameshift altered the amino acid sequence immediately following the edit site and frequently introduced premature termination codons (PTCs; UAA, UAG, or UGA) within the shifted reading frame. Consequently, the resulting transcripts are predicted to encode severely truncated polypeptides lacking all essential functional domains downstream of the cleavage site. Consistent with this mechanism, molecular characterization revealed shorter amplification products and a complete loss of conserved functional motifs in edited sequences. Given that the editing site resides near the N-terminus of the *Vg* coding sequence, the predicted truncations are expected to abolish protein stability and function, either through the production of non-functional polypeptides or via degradation of PTC-containing transcripts by nonsense-mediated mRNA decay (NMD) pathways. These molecular outcomes substantiate the high likelihood of eliciting robust loss-of-function phenotypes while minimizing the risk of in-frame “escape” variants that could retain partial biological activity.

**Table 3:** Main domain mapping of honey bee *Vitellogenin* protein by SMART.

Domain		Start	End	E-value	Feature	GO function
LPD_N		22	741	1.41E-249	Lipoprotein N-terminal domain	Lipid transporter activity
DUF1943		773	1043	2.31E-30	-	Lipid transporter activity
VWD		1435	1614	2.79E-17	Von Willebrand factor (type D domain)	Receptor-mediated oocyte uptake Ligand-binding versatility Immune recognition

### Protein structure prediction

Computational protein modeling predicts that frameshift mutations in exons 2 and 3 result in premature termination and loss of the conserved von Willebrand factor D domain, which is critical for lipid transport and immune function in worker bees.

## Discussion

### Strategic target selection and the pleiotropic architecture of vitellogenin

The strategic selection of target loci remains a critical determinant of success in CRISPR-Cas9–mediated genome editing, particularly when interrogating complex phenotypic traits. Within the omnigenic framework, complex traits are shaped by vast regulatory networks wherein thousands of peripheral genes exert subtle, buffered effects, while a smaller subset of core genes directly drives key biological pathways. Consequently, prioritizing core or main regulatory genes with direct relevance yields substantially higher editing efficiency and phenotypic penetrance than targeting peripheral loci, which are frequently constrained by network redundancy and exhibit minimal individual effect sizes^62–64^. The omnigenic architecture cautions against assuming that GWAS signals, particularly those pointing peripheral genes, represent optimal editing targets^65^. This framework acknowledges the biological reality of omnigenic architecture while maintaining a pragmatic focus on actionable targets with high benefit-to-risk ratios^66^.

In the context of social insect biology, *Vg* in *Apis mellifera* exemplifies a quintessential core gene that operates at the nexus of multiple regulatory networks. Originally characterized for its ancestral role in yolk provisioning during oogenesis^4,67^, *Vg* has been co-opted during honey bee evolution to regulate diverse functions beyond reproduction^4,10,68^, including foraging behavior^68^, lifespan^69,70^, immunity and pathogen defense^5,8,71^, stress resistance^3,72^ and social task allocation within the colony. Targeting *Vg* aligns precisely with the core-gene prioritization strategy advocated by the omnigenic model; however, editing such a highly connected hub necessitates careful consideration of downstream network perturbations. Disruption of *Vg* is expected to propagate beyond its canonical lipoprotein functions, potentially altering transcriptional cascades and physiological trade-offs that are normally buffered under natural conditions. While this pleiotropic architecture presents challenges for predicting isolated phenotypic outcomes, it simultaneously offers a powerful experimental lever for dissecting the molecular underpinnings of complex social traits. Our approach underscores that strategic CRISPR targeting of evolutionarily conserved hub genes can effectively unravel gene-to-phenotype mappings in polygenic systems, provided that validation frameworks account for both direct mechanistic roles and broader network-level consequences. Future investigations should integrate multi-omics profiling to monitor compensatory pathway activation and quantify the extent of pleiotropic trade-offs following targeted disruption, thereby refining predictive models for genome editing in complex regulatory networks.

### Target exons selection rationale

Building on prior evidence that functional deletions and phenotypic markers map to exons 2, 4, and 7 of the *Vg* gene^7^. Therefore, in this study, functional exons were examined computationally to find a suitable target site for CRISPR editing. Among them, the first-order target sites in exons 4 and 7 showed poor efficiency or contained mismatches and off-targets, therefore, they eliminated from further analysis.

Meanwhile, exons 2 and 3 were prioritized for CRISPR/Cas9 targeting based on functional domain mapping, positional efficiency for loss-of-function induction, and the feasibility of gRNA design. Additionally, exons 2 and 3 reside within the first ~ 15% of the coding sequence, ensuring that frameshift indels efficiently trigger nonsense-mediated decay and produce severely truncated polypeptides. Functionally, exon 2 encodes the lipid-binding cavity of the N-terminal β-sheet, while exon 3 contains the N-terminal portion of the VWD domain; disruption of either module disrupts distinct aspects of *Vg*’s dual role in lipid transport and immune recognition. This strategy maximizes the probability of generating a true null allele while enabling modular phenotypic dissection.

### Structural accessibility of target sites

RNA secondary structure analysis revealed marked differences in the biostructure properties of the two lead target sites. The gRNA targeting exon 2 exhibited substantially weaker secondary structure formation, characterized by a higher (less negative) ensemble free energy (ΔG = –0.25 kcal/mol) compared to gRNA targeting exon 3 (ΔG = –2.10 kcal/mol). This reduced structural constraint at the target site of exon 2 is predicted to enhance Cas9 ribonucleoprotein accessibility to the protospacer sequence, a feature empirically associated with 2–5 × higher editing efficiency in prior studies^30,60,73,74^. Ensemble diversity metrics further differentiated the sites for example; gRNA targeting exon 2 displayed lower conformational heterogeneity (1.45 versus 2.26 for gRNA targeting exon 3), indicating a more constrained structural ensemble with reduced competing fold populations that might otherwise occlude the target sequence during critical windows of Cas9 engagement. Both sites exhibited dominant minimum free energy (MFE) structures representing > 60% of the ensemble, with gRNA targeting exon 2 showing marginally higher MFE frequency (66.28%), collectively suggesting predictable folding behavior favorable for reproducible editing outcomes.

### Functional disruption potential

InDelphi-based indel profiling predicted divergent frameshift potentials between the two target sites of exon 2 and 3. The examined target site of exon 3 achieved a frameshift frequency of 88.3% (95.2 percentile), classified as very high disruptive potential, whereas examined target site of exon 2 yielded 77.8% (61.9 percentile), representing moderate potential. The predominant indel classes, 8-bp and 11-bp deletions alongside 1-bp insertions, collectively comprised 88% and 78% of predicted outcomes for target sites of exons 3 and 2, respectively. Critically, non-frameshift (in-frame) alterations accounted for only 12% of edits at the exon 3 site versus 22% at exon 2, substantially reducing the probability of functional escape variants that might retain partial vitellogenin activity. These profiles indicate a high likelihood of complete loss-of-function at both target sites, that the targeted site of exon 3 offering superior assurance of reading frame disruption essential for interrogating vitellogenin’s pleiotropic roles in reproduction, immunity, and social physiology.

### Integrated interpretation and experimental implications

The complementary biophysical and functional profiles of these target sites present a strategic trade-off inherent to CRISPR design for pleiotropic regulators, as a result the predicted editing outcome for the gRNA targeting exon 2 maximizes structural accessibility, favoring higher absolute editing rates through enhanced Cas9 binding, whereas the predicted editing outcome for the gRNA targeting exon 3 maximizes functional disruption completeness, favoring near-certain loss-of-function among successfully edited alleles. This dichotomy reflects a fundamental tension in genome editing that potentially maximal on-target cutting does not invariably translate to maximal phenotypic impact when in-frame repair products retain partial function. Given the logistical constraints of honey bee embryo microinjection and the irreversibility of G0 editing in haplodiploid systems, we recommend parallel empirical validation of both sites. Such a dual-target strategy accommodates uncertainty in the relative weighting of cutting efficiency versus frameshift completeness under physiological conditions while maximizing the probability of generating informative loss-of-function phenotypes. Our integrated in silico framework demonstrates how computational pre-screening, combining structural thermodynamics with functional outcome prediction, may substantially reduce experimental attrition in non-model organisms where wet-lab optimization incurs disproportionate biological and resource costs. This approach establishes a transferable template for rational CRISPR design targeting pleiotropic master regulators across complex genomes.

### Structural impact of frameshift editing on *Vitellogenin*

**Fig. 8:**
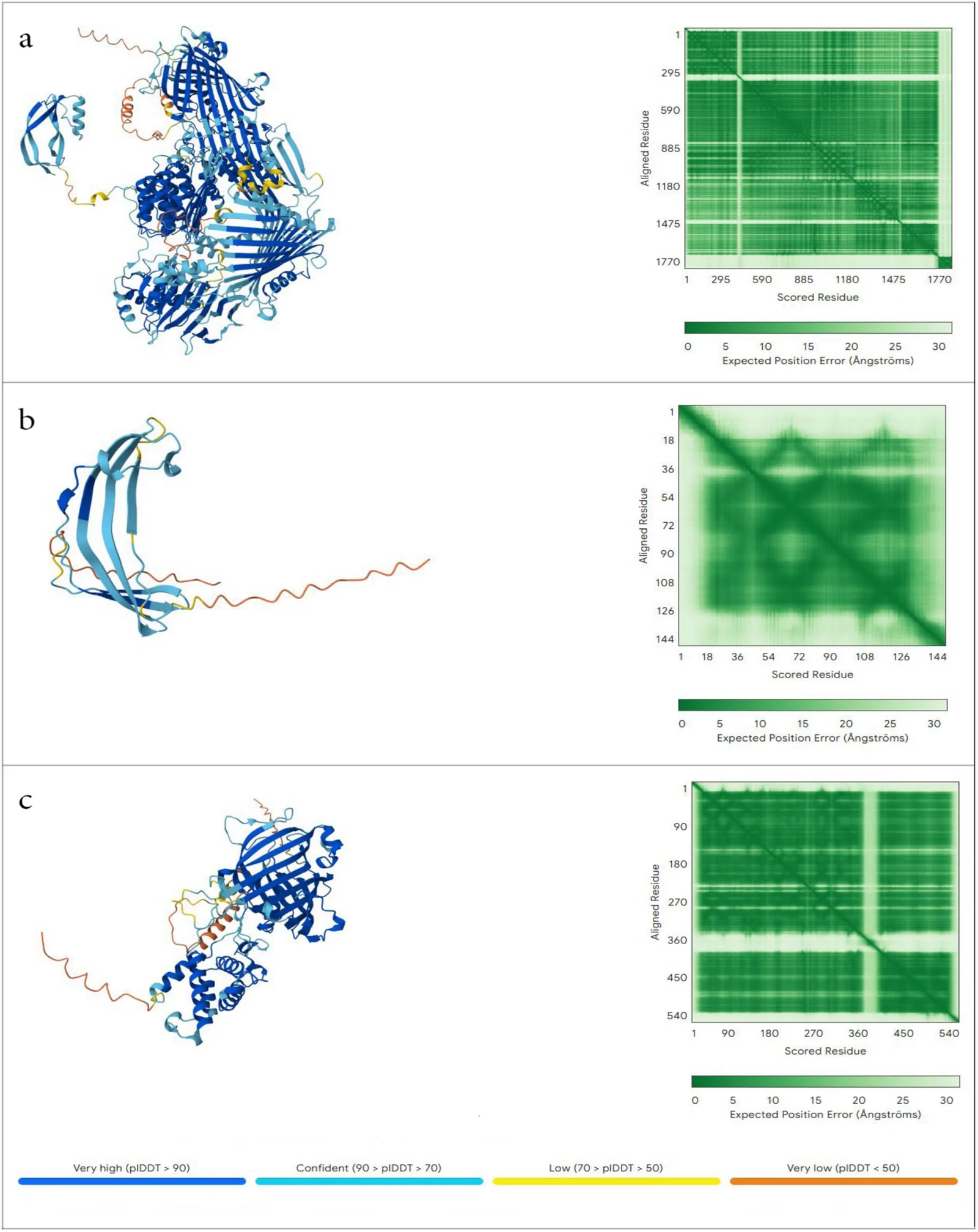
Predicted protein structure of wild type Vg (**a**), frameshift edit targeting exon 2 (**b**), and frameshift edit targeting exon 3.

AlphaFold modelling (Fig. 8) of the wild-type *vitellogenin* (*Vg*) protein yielded a high-confidence structure (mean pLDDT: 0.86), clearly resolving the canonical N-terminal β-sheet domain, central α-helical region, polyserine linker, DUF1943 module, and C-terminal VWD domain. In silico insertion of a single nucleotide in targeting site of exon 2 induced a + 1 frameshift that altered all downstream codons and introduced a premature termination codon at residue 145. The resulting truncated polypeptide lacks all downstream domains, including LPD-N, DUF1943 module, and the C-terminal VWD domains.

A second single-base insertion in predicting targeting site of exon 3 shifted the reading frame and generated a premature stop codon at position 543. This variant preserves the N-terminal region and the LPD-N domain but truncates both the DUF1943 module and the C-terminal VWD domain.

The VWD domain is an evolutionarily conserved, cysteine-rich module that in honeybee *Vg* mediates receptor-dependent oocyte uptake during yolk deposition and contributes to innate immunity through pathogen recognition and agglutination^11,75^. Structural analyses have identified a highly conserved Ca^2^⁺-binding site within this domain, suggesting a role in conformational regulation or ligand coordination^76^. Recent cryo-EM reconstructions of native *Apis mellifera Vg* position the VWD domain distal to the lipid-binding core, supporting its primary function in extracellular receptor interactions rather than direct lipid transport^8^. Consequently, frameshift-mediated loss of the VWD and DUF1943 modules is predicted to abolish both yolk deposition and immune-related functions, providing a clear mechanistic basis for the null phenotype expected in haplodiploid individuals.

Both frameshifted models exhibited a sharp decline in structural prediction confidence beyond the truncation sites (pLDDT < 50), consistent with intrinsically disordered or rapidly degraded C-terminal fragments. Collectively, these computational predictions demonstrate that single-nucleotide insertions in targeting sites of exons 2 and 3 generate severely truncated *Vg* variants lacking critical functional domains, strongly supporting a complete loss-of-function outcome consistent with established principles of translational frameshifts.

### Rationales and limitations

We prioritized SpCas9 for this in silico pipeline because its NGG PAM requirement aligns with rigorously validated NHEJ frameshift predictors (inDelphi and CHOPCHOP), enabling high-confidence knockout screening. Given honeybee haplodiploidy, a single disruptive indel reliably produces null phenotypes without HDR templates, and SpCas9’s well-characterized off-target landscape permits accurate computational filtering. Conversely, PAM-flexible variants (e.g., Cas9-NG) and base editors lack Hymenoptera-calibrated prediction models, introducing unquantified off-target risks, while their delivery in *Apis mellifera* remains unoptimized. SpCas9 guide RNAs also integrate directly with established microinjection and RNP electroporation workflows, ensuring immediate experimental readiness. This framework, however, carries defined constraints including the predictor tools were trained primarily on mammalian and Drosophila datasets, so species-specific DNA repair kinetics may influence empirical outcomes, and the NGG restriction limits targetable loci compared to next-generation editors. Moreover, because honeybee embryo editing typically employs RNP microinjection rather than viral delivery, the size advantage of SaCas9 confers minimal practical benefit in this system. Future work will experimentally validate top-ranked gRNAs and expand computational screening to PAM-relaxed Cas9 orthologs and deaminase-based editors to model *Vg* hypomorphs and caste-specific regulatory variants.

While our computational off-target screening employs three orthogonal predictors and conservative mismatch thresholds, we acknowledge that empirical validation (e.g., GUIDE-seq, CIRCLE-seq, or targeted deep sequencing of predicted off-target loci) remains the definitive benchmark. The current framework is optimized to prioritize low-risk candidates for downstream RNP-based delivery, which empirically suppresses off-target activity. As honeybee-specific off-target datasets expand, integrating organism-specific chromatin state maps and repair kinetics will further refine predictive accuracy.

To advance from in silico simulation to empirical CRISPR/Cas9 editing, we recommend using microinjection of pre-assembled sgRNA:Cas9 ribonucleoprotein complexes, which should be performed within two hours post-oviposition, with precise delivery to the ventral cephalic region of the embryo to maximize germline editing efficiency. Biallelic disruption in G0 somatic tissues is computationally feasible, enabling phenotypic assessment within a single generation, a critical advantage given the logistical constraints of multi-generational breeding in haplodiploid, eusocial insects. The constitutive expression of *Vg* in functionally sterile workers presents a unique opportunity to dissect non-reproductive functions of this yolk protein, including roles in oxidative stress resistance and behavioral regulation, without confounding reproductive physiology. Finally, sgRNA design should prioritize minimal homology to *Vg-like* paralogs to mitigate functional compensation within the expanded *Apis mellifera* vitellogenin gene family.

## Supplementary Information

Supplementary Information.

## Data Availability

All the data are inside the manuscript.
